# Malaria Care in Infants Aged under Six Months in Uganda: An Area of Unmet Needs!

**DOI:** 10.1371/journal.pone.0123283

**Published:** 2015-04-10

**Authors:** Martin Kayitale Mbonye, Sarah M. Burnett, Sarah Naikoba, Robert Colebunders, Kristien Wouters, Marcia R. Weaver, Jean Pierre Van Geertruyden

**Affiliations:** 1 Infectious Diseases Institute, Makerere University, Kampala, Uganda; 2 Department of Scientific Coordination and Biostatistics, Antwerp University Hospital, Antwerp, Belgium; 3 Accordia Global Health Foundation, Washington DC, United States of America; 4 Unit of International Health, Faculty of Medicine and Health Sciences, University of Antwerp, Antwerp, Belgium; 5 Department of Clinical Sciences, Institute of Tropical Medicine, Antwerp, Belgium; 6 International Training and Education Center for Health (I-TECH), Department of Global Health, University of Washington, United States of America; Kliniken der Stadt Köln gGmbH, GERMANY

## Abstract

**Background:**

Little information exists on malaria burden, artemisinin-based combination therapy (ACT) use, and malaria care provided to infants under six months of age. The perception that malaria may be rare in this age group has led to lack of clinical trials and evidence-based treatment guidelines. The objective of this study was to identify malaria parasitemia positivity rate (MPPR) among patients under six months, and practices and predictors of malaria diagnosis and treatment in this population.

**Methods:**

Cross-sectional data collected from October 2010 to September 2011 on 25,997 individual outpatients aged <6 months from 36 health facilities across Uganda were analysed.

**Findings:**

Malaria was suspected in 18,415 (70.8%) patients, of whom 7,785 (42.3%) were tested for malaria. Of those tested, the MPPR was 36.1%, with 63.9% testing negative, of which 1,545 (31.1%) were prescribed an antimalarial. Among children <5kgs, off-label prescription of ACT was high (104/285, 36.5%). Younger age (1-6 days, aOR=0.47, p=0.01; 7-31 days, aOR=0.43, p<0.001; and 1-2 months, aOR=0.61, p<0.001), pneumonia (aOR=0.78, p=0.01) or cough/cold (aOR=0.65, p<0.001) diagnosis, and fever (aOR=0.56, p=0.01) reduced the odds of receiving a malaria test. Fever (aOR=2.22, p<0.001), anemia diagnosis (aOR=3.51, p=0.01), consulting midwives (aOR=3.58, p=0.04) and other less skilled providers (aOR=4.75, p<0.001) relative to medical officers, consulting at hospitals (aOR=3.31, p=0.03), visiting health facilities in a medium-high malaria transmission area (aOR=2.20, p<0.001), and visiting during antimalarial (aOR=1.82, p=0.04) or antibiotic (aOR=2.23, p=0.04) shortages increased the odds of prescribing an antimalarial despite a negative malaria test result.

**Conclusions:**

We found high malaria suspicion but low testing rates in outpatient children aged <6 months. Among those tested, MPPR was high. Despite a negative malaria test result, many infants were prescribed antimalarials. Off-label ACT prescription was common in children weighing <5kgs. Evidence-based malaria guidelines for infants weighing <5 kilograms and aged <6 months are urgently needed.

## Background

In a recent review on malaria in infants aged under six months, D’Alessandro *et al*. concluded that the perception that malaria was rare in this population had been responsible for the lack of evidence and research on treatment guidelines regarding this population. They opined that malaria in this age group may not be rare after all and that its burden may be underestimated, especially in endemic countries [1]. Despite recent efforts and successes in the fight against malaria [2–5], the disease remains the leading cause of morbidity and mortality, with an estimated 198 million clinical episodes and 584,000 deaths reported globally in 2013 [6]. Approximately 78% of these deaths occurred in children under five years of age and 90% occurred in the WHO Africa Region [6]. In Uganda, malaria is the leading cause of mortality among children under five, accounting for 32% and 17.5% of all deaths in this age group in 2007 [7] and 2010 [8] respectively. According to the 2007 verbal autopsy results, malaria was responsible for 41% of all post neonatal deaths [7]. Among children under one, malaria causes approximately 545 deaths per 100,000 population, higher than 190 per 100,000 estimated for children aged one to five years old [8].

Previous studies concluded that young infants aged under six months were protected against malaria—making malaria in this group a rare phenomenon, even though mechanisms for the apparent protection were not clearly understood [9,10]. Initially, this protection was thought to be due to passively acquired maternal immunoglobulin G (IgG) antibodies [11–13]. Demonstration of parasite growth inhibitory characteristics *in vitro* by lactoferrin and immunoglobin A (IgA) found in breast milk and maternal and infant sera has led others to hypothesize that the protection is associated with these factors [12–14]. It is also documented that hemoglobin F (HbF), which is present in high concentrations at birth inhibits parasite development and can protect an infant in the first few months of life [11,15–17]. However, some studies have failed to demonstrate the relationship between presence of maternal antibodies and protection against malaria in young African infants. Instead, presence of maternal antibodies appeared to correlate with a higher risk of exposure to malaria infection [17,18].

A few recent reports show that malaria in infants aged under six months may not be a rare occurrence and that its burden may be underestimated [1,19,20]. Otherwise information on the impact of malaria in this population is limited. In Uganda and Kenya, where an analysis of malaria prevalence in this age group was done as a part of the National Malaria Indicator Surveys, the malaria prevalence was reported to be 16% and 5%, respectively [21,22].

Despite growing information regarding the potentially high prevalence of malaria in infants aged under six months, strong clinical evidence regarding how best to treat these cases is lacking. Conflicting data on the prevalence, coupled with ethical and practical considerations for implementing clinical trials among this age group, have led to a gap in evidence-based treatment guidelines [23]. While the World Health Organization (WHO) recommends ACTs as first-line treatment for infants and young children with uncomplicated malaria, they state that this is not a “confident recommendation” for infants under 5 kilograms (<5kgs) [23]. In Uganda, quinine, rather than ACT, is recommended for treatment of malaria in children below four months of age or five kilograms (<5kgs) of weight [24]. Younger infants may however be more susceptible to quinine toxicity than older children [25,26]. The pharmacokinetic profile of antimalarials, such as quinine and ACTs, may also be different in young infants than it is in older children due to dynamic developmental changes, making age-dependent dosing necessary [27]. Although ACTs carry label restrictions for this age group [23], clinicians often prescribe ACTs off-label based on the recommended milligrams per kilogram dosing schedule for older children [20].

Research on the burden of malaria, ACT use, and predictors of malaria care in primary care settings for infants under six months is needed to inform evidence-based guidelines for malaria care in this population. We report findings from a secondary analysis of facility data on 25,997 outpatient visits for infants aged under six months collected during the Integrated Infectious Diseases Capacity Building Evaluation (IDCAP) [28–30]. The objective of this study was to identify the malaria parasitemia positivity rate (MPPR) among malaria suspects aged under six months, and practices and predictors of malaria diagnosis and treatment in this population.

## Methods

### Ethics statement

The original IDCAP protocol was reviewed and approved by the School of Medicine Research and Ethics Committee of Makerere University and the Uganda National Council for Science and Technology. Data used in this analysis were collected for the health management information system (HMIS) as part of routine reporting at health facilities and did not require informed consent. The University of Washington Human Subjects Division determined that IDCAP did not meet the regulatory definition of research under 45 CFR 46.102(d). This secondary analysis of anonymous data was exempt from review by the University of Antwerp ethical review board. The anonymous data used in this analysis and instructions for requesting them are available on the Global Health Data Exchange website at http://ghdx.healthdata.org/node/176574.

### Study design and setting

Data were collected from a convenience sample of 36 health center IVs (HCIVs) or comparable health facilities. HCIVs are the highest healthcare referral point for a health sub-district [31,32] and each HCIV is expected to serve a population of about 100,000 people and provide basic preventive, curative and referral services. They have limited inpatient wards and conduct some emergency and surgical and obstetric procedures [31,33]. Staffing norms and level of staffing for a HCIV are reported in the 2010 Ministry of Health Human Resources for Health Audit Report [34].

The facilities represented 28 of 114 current districts. As shown on the map of Uganda with the location of these health facilities [35], they represented all major regions of Uganda and were widely distributed across the country. One inclusion criterion for facilities was presence of a functional laboratory that could conduct the following six investigations: HIV rapid test, malaria blood smear, TB sputum smear, urinalysis, stool analysis, and hemoglobin estimation. One exclusion criterion was actively participating in the national quality improvement programs for HIV/AIDS services. To the extent that these national programs selected the most accessible facilities, the IDCAP facilities may have been more remote than other HCIVs. Other inclusion and exclusion criteria were described in detail in Naikoba *et al*. and Miceli *et al*. [28,36].

Cross-sectional data were collected prospectively from November 2009 to September 2011 using a standardized outpatient medical form (MF5), initially designed by Uganda Malaria Surveillance Project [37] and revised by IDCAP as described in Mbonye *et al*. [30,35]. This secondary analysis focused on the final year (October 2010 to September 2011), because the quality of data improved over time from on-going technical support. The data collection system was described in detail in Mbonye *et al*. [30].

### Study participants

All outpatients aged under six months that visited the 36 health facilities during the 12-month study period participated as part of their routine process of care.

### Definitions

A patient suspected with malaria was defined as a patient visit with a fever or a history of fever, referral for malaria laboratory testing, or malaria diagnosis (clinical or parasitological).

A clinical diagnosis of malaria was defined as a patient visit with a record of malaria diagnosis and/or an antimalarial prescription without a malaria test, or with an antimalarial prescription when the test result was negative.

Parasitological diagnosis of malaria was defined as a patient visit with a positive diagnostic test result for malaria recorded.

An appropriate antimalarial referred to a prescription for first or second line treatment recorded and included quinine and the following ACTs: artemether & lumenfantrine, artesunate & amodiaquine, or dihydroartemisinin & piperaquine phosphate (Duocotecxin) [24].

Any antimalarial treatment referred to a prescription for an appropriate antimalarial listed above or any of the three antimalarial drugs that did not comply with the Uganda National guidelines [24]: amodiaquine alone, chloroquine, and sulfadoxine/pyrimethamine (SP) (Fansidar).

Malaria parasitemia positivity rate (MPPR) was defined as the ratio of laboratory-confirmed malaria patients to malaria suspects tested.

### Outcomes

The outcomes were MPPR as defined above, and three malaria care variables: 1) Proportion of patients with suspected malaria for whom a diagnostic test result for malaria was recorded, 2) Proportion of patients with a negative test result for malaria who were prescribed any antimalarial and 3) Proportion of patients ≥5kgs prescribed an appropriate antimalarial among those prescribed any antimalarial. Data used to classify patients for each outcome were recorded on the MF5. Note that the Ugandan national policy on malaria treatment does not recommend submitting blood samples for polymerase chain reaction (PCR) tests to confirm a negative malaria test result as part of routine care at HCIV.

### Explanatory variables

Eighteen explanatory clinical and operational variables that may have influenced a provider’s decision on how to manage a patient were selected for the analysis. Patient-level clinical explanatory variables were triage status, weight for age, age group, fever, number of diagnoses and the following diagnoses commonly associated with fever in young children: cough or cold, diarrhea, pneumonia, urinary tract infections and anemia. Patient-level operational explanatory variables were patient’s return visit with the same chief complaint as the previous visit and health provider’s level of training. Facility level operational explanatory variables were entomological inoculation rate (EIR) of the area surrounding the health facility, health facility level, health facility type, staffing and stock outs for three drugs: antimalarials, antibiotics, and oral rehydration salts (ORS).

Triage status was defined according to the World Health Organization (WHO) Emergency Triage, Assessment and Treatment (ETAT) guidelines [38] and categorised by health providers on the MF5 as emergency, priority or standard. Weight was categorised as normal or underweight for age using WHO z-scores for child growth standards [39]. Age was categorised into seven levels including: 1–6 days, 7–31 days, 1–2 months, 2–3 months, 3–4 months, 4–5 months and 5–6 months. We relied on the diagnosis decision of the clinician as reflected by the tick on the checkboxes on the MF5 for illnesses, such as cough/cold (no pneumonia), and pneumonia. Number of diagnoses was categorized into three levels as no diagnosis, single diagnosis or multiple diagnoses according to the number of illnesses recorded on the MF5. The MoH refers to EIR as the annual number of mosquito infective bites per person. EIR was categorized as very low (<1), low (1–10), medium- high (11–100) and very high (>100) mosquito infective bites per person per year respectively [40]. The two staffing variables were categorized in quartiles: 1) clinically active staff quartile calculated as the number of clinical staff who saw at least five patients during a month as a proportion of patients at the facility during that month and 2) lab staff quartile calculated as laboratory professionals assigned to the facility in February and March 2010 as a proportion of the ideal where ideal was defined by Uganda Ministry of Health staffing norms [41]. Availability rate of antimalarials, antibiotics and ORS drugs was calculated by dividing the actual number of patients who received the drug with the number of patients who were prescribed the drug in question. Shortage rates for each of these drugs were calculated for each week by subtracting the availability rate of each drug during that week from 100%. Health provider was categorized in order of decreasing years of training including medical officer, clinical officer, nurse, midwife and other less skilled providers. Level of health facility was categorized as health center IV or small hospital. Type of health facility was categorized as government or private-not-for-profit (PNFP).

### Statistical analysis

This is a multilevel analysis of cross-sectional data from all health facilities that implemented IDCAP. Descriptive statistics, specifically frequencies and percentages, were generated to describe the patient population. Logistic regression was used to evaluate univariate relationships between the explanatory variables and each outcome. Only the explanatory variables that displayed significant associations with the outcome at p<0.10 in the univariate analysis were included in the multivariate analysis. The multivariate regression model used generalized estimating equations and clustered by health facility to account for intra-health facility correlation. A link test was used in the univariate analyses and both link test and Receiver Operator Characteristic (ROC) curves were used in the multivariate analyses to assess model fit. In the multivariate models, a 5% level of significance was used to test whether the association between explanatory variables and the outcomes were statistically significant and the results are presented with 95% confidence intervals (CI). All analyses were performed using Stata version 12 (Stata Corp, College Station TX, USA).

In addition to the primary explanatory variables, the variable month of the patient visit (month) was identified *a priori* as a potential extraneous factor, given the seasonal nature of malaria and was included and controlled for in the multivariate model when associated with the outcome of interest in univariate analysis (p<0.10).

Three key exploratory variables that were statistically significant at univariate analysis had missing data: provider-training level (12%), return visits (3.5%) and fever (26.4%). Multiple imputation analysis was done to address missing values for these three variables. Multivariable imputation using chained equations (MICE) was used on the assumption that these data were missing at random. Logistic regression was then used to impute missing values with five iterations and estimates derived from each iteration were combined using Rubin’s methods [42]. Although weight-for-age and triage status had missing data, they were not statistically significant at univariate analysis and were thus not considered for multiple imputation analysis at multivariate level.

A sensitivity analysis was done using only complete cases and the estimates did not substantially differ from those produced when using multiple imputation. Only estimates of odds ratios (OR), 95% confidence intervals (CI) and p-values derived through multiple imputation analysis are presented. Potential influence of the IDCAP interventions was not controlled for since the data used were collected towards the end of the trial in December 2010 and during follow-up when both arms received the trial intervention.

## Results

A total of 25,997 infants under six months visited the 36 health facilities during 12 months from October 2010 to September 2011. Table 1 reports the characteristics of these patients. Of these, 3,472 (13.4%) were neonates defined as under one month old, 25,474 had complete data on gender of which 12,994 (51.0%) were males, and 22,630 (87.1%) were triaged, of which 729 (3.2%) required emergency attention.

**Table 1:** Characteristics of the infant patients under the age of six years Ugandan Primary Health centers.

Characteristics/Parameters	n (25,997)	%
Female	12,480	49.0%
Age group		
1–6 days	918	3.5%
7–31 days	2,554	9.8%
1–2 month	3,632	14.0%
2–3 months	3,870	14.9%
3–4 months	4,557	17.5%
4–5 months	5,113	19.7%
5–6 months	5,353	20.6%
Triage status		
Standard triage status among triaged	19,577	86.5%
Priority triage status among triaged	2,324	10.3%
Emergency triage status among triaged	729	3.2%
> = 5kgs among patients with weight	8,296	79.2%
*Weight for age		
Normal weight for age	9,240	89.9%
Underweight for age	965	10.4%
Severely underweight for age	71	7.4%
History of Fever (All)	15,691	82.0%
Fever (thermometer: Temp>37.5°C)	988	5.2%
^£^Cough or cold diagnosis	8,189	31.5%
^£^Diarrhea diagnosis	2,664	10.2%
^£^Pneumonia diagnosis	3,373	13.0%
^£^Urinary tract infection diagnosis	1,077	4.1%
^£^Anemia diagnosis	268	1.0%
Number of diagnoses		
With multiple diagnosis	12,557	48.3%
With a single diagnosis (any illness)	11,792	45.4%
With no diagnosis	1,648	6.3%
Prescribed an antimalarial	10,134	39.0%
Prescribed Antibiotics	19,712	75.8%
Prescribed Multivitamins	201	0.8%
Prescribed Oral Rehydration Salts (ORS)	4,096	15.8%
Admitted	2,723	10.5%
Referred	445	1.7%
Detained for monitoring	70	0.3%
Repeat visit	487	1.9%
Antimalarial unavailability (> = 50% of the time)	1,670	21.3%
Antibiotic unavailability (> = 50% of the time)	5,755	38.2%
ORS unavailability (> = 50% of the time)	709	22.7%
EIR		
Very low EIR area	1,429	5.5%
Low EIR area	1,471	5.7%
Medium—high EIR area	3,634	14.0%
Very high EIR area	19,463	74.9%
Health provider		
Medical officer	452	1.9%
Clinical officer	13,115	55.6%
Nurse	6,273	26.6%
Midwife	200	0.8%
Other (less skilled)	2,904	12.3%
HCIV	21,828	84.0%
Government	23,394	90.0%

*Weight for age was based on WHO z-scores for child growth standards and it was therefore calculated for patients with sex & weight data.

^£^ Commonly diagnosed illnesses associated with fever.

Weight was recorded for only 10,477 (39.5%) patients of whom 965 (10.4%) were underweight. Fever or history of fever was assessed in 19,124 (73.6%) patient visits, among which fever was reported in 15,691 (82.0%) and absence of fever in 3,433 (18.0%) of the patient visits. Cough or cold, pneumonia, diarrhea, urinary tract infection or anemia, illnesses frequently associated with fever, were diagnosed in 31.5%, 13.0%, 10.2%, 4.1% and 1.0% patients respectively. Of the 25,997 patients seen, 12,557 (48.3%) had multiple diagnoses; 10,134 (39.0%), 19,712 (75.8%) and 4,096 (15.8%) were prescribed antimalarials, antibiotics and ORS respectively.

In addition, 481 (1.9%) of the 25,081 patients with a record of the visit type were return visits for the same chief complaint. A total of 19,363 (74.9%) patients visited health facilities in very high EIR areas, 21,828 (84.0%) patients visited HCIVs, and 23,394 (90.0%) visited government health facilities. Of the 22,944 (88.3%) patient visits with health provider data recorded, the majority were managed by clinical officers (13,155; 55.6%) and nurses (6,273; 26.6%). Antibiotics, antimalarials and ORS were unavailable more than 50% of the time during 38.2%, 21.3% and 22.7% of the patient visits, respectively.

### Diagnostic testing for malaria among patients with suspected malaria

**Fig 1:**
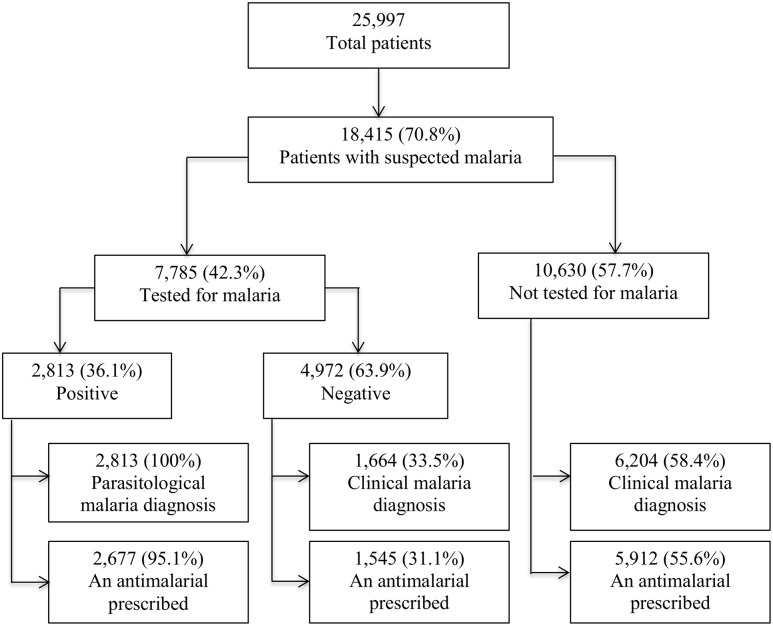
Flow chart for malaria diagnosis in young infants in 36 Ugandan Primary Health centers, October 2010 – September 2011.

**Fig 2:**
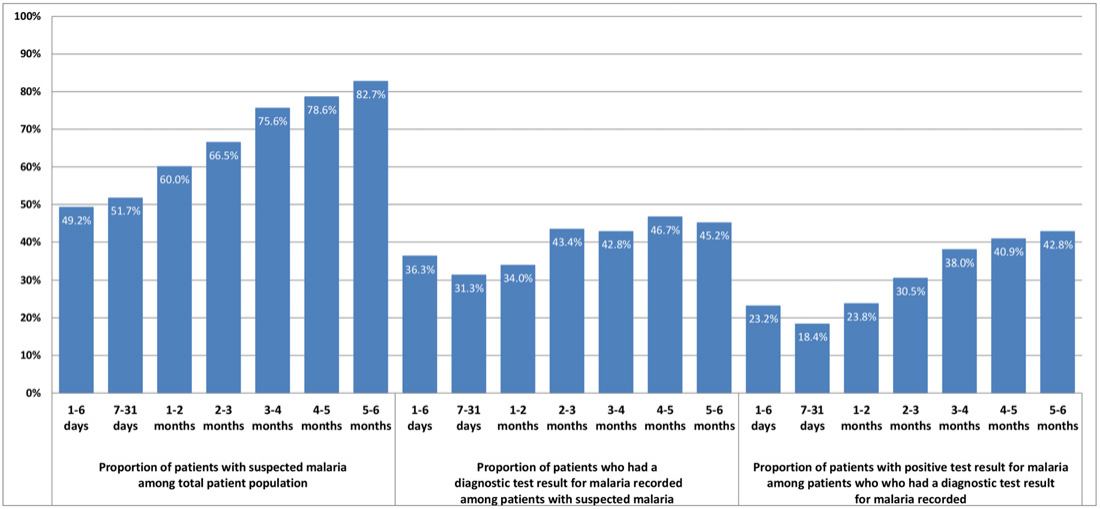
Malaria suspicion, testing and parasitemia positivity rates by age group in infants aged less than six months in Ugandan Primary Health centers, October 2010 – September 2011.

**Fig 3:**
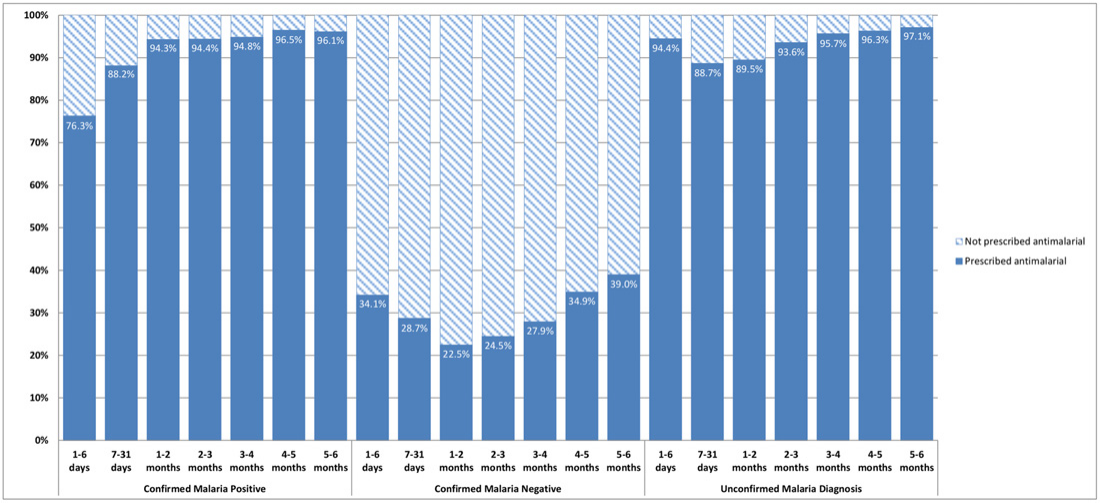
Malaria parasitemia positivity rate by month for infants aged less than six months in Ugandan Primary Health centers, October 2010 – September 2011.

As shown in Fig 1, of the 25,997 visits, 18,415 (70.8%) were suspected with malaria but only 7,785 (42.3%) of these had a diagnostic test result for malaria recorded. MPPR among patient visits with a diagnostic test result for malaria was 36.1%. The MPPR was under 24% in all the three age groups for infants aged under two months (Fig 2). The MPPR was highest in November 2010 (41.6%) and lowest in March 2011 (30.2%), which corresponds to rainy and dry seasons in much of Uganda (Fig 3). Of the 4,972 patients with confirmed negative diagnostic test result for malaria, 1,664 (33.5%) also had a clinical diagnosis of malaria. Among the 10,630 patients with suspected malaria who did not have a diagnostic test result for malaria, 6,204 (58.4%) had a clinical diagnosis of malaria.

In the univariate analysis (Table 2), patients with suspected malaria were less likely to have a diagnostic test result for malaria recorded if they were in a younger age group (1–6 days, 7–31 days, 1–2 month or 3–4 months relative to age group 5–6 months); had a fever; had a cough or cold or pneumonia diagnosis; were return visitors; visited a hospital; were seen by a clinical officer, nurse, midwife or other less skilled provider relative to a medical officer or visited during the weeks the health facility faced shortages of antimalarials, antibiotics or ORS. They were, on the other hand, more likely to have a diagnostic test result for malaria recorded if they had no diagnosis relative to single diagnosis; had a diarrhea or anemia diagnosis; visited a PNFP health facility; visited a health facility in medium—high EIR or low EIR relative to those in very high EIR areas or visited a health facility with clinically active or laboratory staff in 2^nd^, 3^rd^ and highest quartiles relative to the lowest quartile.

**Table 2:** Predictors of a patient with suspected malaria with a diagnostic test result for malaria recorded in Ugandan Primary Health centers.

Variable	Patients with suspected malaria		Univariate analysis		Multivariate Analysis	
	Total (N = 18,415)	% With diagnostic test result for malaria recorded (n = 7,785)	cOR (95% CI)	p Value	aOR (95% CI)	p Value
Age group						
1–6 days	452	36.3%	0.69 (0.57, 0.84)	<.0001	*0.47 (0.27, 0.85)	0.01
7–31 days	1,321	31.3%	0.55 (0.49, 0.63)	<.0001	*0.43 (0.29, 0.63)	<0.0001
1–2 months	2,181	34.0%	0.63 (0.56, 0.70)	<.0001	*0.60 (0.51, 0.72)	<0.0001
2–3 months	2,572	43.4%	0.93 (0.85, 1.03)	0.16	0.90 (0.78, 1.04)	0.16
3–4 months	3,444	42.8%	0.91 (0.83, 0.99)	0.04	0.90 (0.79, 1.02)	0.09
4–5 months	4,018	46.7%	1.06 (0.98, 1.16)	0.16	1.03 (0.96, 1.11)	0.42
5–6 months	4,427	45.2%	*ref*	*Ref*	*ref*	*Ref*
Fever	15,691	41.0%	0.70 (0.65, 0.76)	<.0001	*0.56 (0.36, 0.88)	0.01
Number of diagnoses						
Single diagnosis	6,162	41.8%	*ref*	*Ref*	*ref*	*Ref*
Multiple diagnosis	11,448	41.6%	0.99 (0.93, 1.06)	0.82	0.92 (0.71, 1.19)	0.53
No Diagnosis	805	56.2%	1.79 (1.54, 2.07)	<.0001	*1.38 (1.07, 1.78)	0.01
Cough or cold diagnosis	5,594	39.6%	0.85 (0.80, 0.91)	<.0001	*0.78 (0.66, 0.93)	0.01
Diarrhea diagnosis	2,092	47.7%	1.28 (1.17, 1.40)	<.0001	1.18 (0.99, 1.40)	0.06
Pneumonia diagnosis	2,619	34.8%	0.69 (0.64, 0.75)	<.0001	*0.65 (0.53, 0.81)	<0.0001
Anemia diagnosis	245	53.5%	1.57 (1.23, 2.03)	0.001	1.32 (0.85, 2.06)	0.21
Return visit	300	29.3%	0.57 (0.44, 0.73)	<.0001	0.46 (0.18, 1.17)	0.96
Health provider						
Medical officer	272	50.4%	*ref*	*ref*	*ref*	*ref*
Clinical officer	9,177	41.9%	0.71 (0.56, 0.91)	0.01	1.14 (0.57, 2.27)	0.71
Nurse	4,457	40.7%	0.68 (0.53, 0.86)	0.002	1.04 (0.51, 2.10)	0.92
Midwife	127	39.4%	0.64 (0.42, 0.98)	0.04	1.05 (0.42, 2.65)	0.91
Other (less skilled)	2,206	43.6%	0.76 (0.59, 0.98)	0.03	1.02 (0.47, 2.20)	0.96
EIR area						
Very high	14,667	37.4%	*ref*	*ref*	*ref*	*Ref*
Medium-high	2,405	71.0%	4.09 (3.73, 4.50)	<.0001	*3.04 (1.59, 5.78)	0.001
Low	360	46.8%	1.47 (1.27, 1.70)	<.0001	1.29 (0.56, 2.95)	0.55
Very low	573	40.7%	1.15 (0.97, 1.36)	0.11	0.78 (0.23, 2.61)	0.69
Clinically active staff quartile						
Lowest	5,048	35.8%	*ref*	*Ref*	*ref*	*Ref*
2^nd^ quartile	5,056	41.0%	1.25 (1.15, 1.35)	<.0001	*1.33 (1.01, 1.77)	0.05
3^rd^ quartile	4,954	50.5%	1.45 (1.34, 1.57)	<.0001	*1.72 (1.23, 2.39)	0.001
Highest	3,357	42.3%	1.83 (1.67, 2.00)	<.0001	*1.88 (1.25, 2.82)	0.002
Lab staff quartile						
Lowest	6,325	28.1%	*ref*	*Ref*	*ref*	*Ref*
2^nd^ quartile	4,684	52.1%	2.79 (2.57, 3.02)	<.0001	*2.58 (1.45, 4.62)	0.001
3^rd^ quartile	5,016	45.1%	2.14 (1.98, 2.32)	<.0001	*1.60 (1.04, 2.44)	0.03
Highest	2,390	53.9%	2.99 (2.72, 3.30)	<.0001	1.77 (0.73, 4.27)	0.21
Hospital	3,028	38.7%	0.84 (0.78, 0.91)	<.0001	0.63 (0.39, 1.00)	0.05
Private Not for Profit	1,663	59.4%	2.14 (1.93, 2.38)	<.0001	1.18 (0.37, 3.81)	0.78
Antimalarial shortage	3,710	31.7%	0.57 (0.53, 0.62)	<.0001	1.33 (0.74, 2.41)	0.34
Antibiotic shortage	8,283	39.1%	0.79 (0.75, 0.84)	<.0001	0.95 (0.63, 1.44)	0.81
ORS shortage	5,156	34.7%	0.65 (0.60, 0.69)	<.0001	0.54 (0.28, 1.06)	0.07

* Statistically significant at 0.05 level.

In multivariate analysis, age groups 1–6 days (aOR 0.47, 95%CI 0.27, 0.85), 7–31 days (aOR 0.43, 95%CI 0.29, 0.63) and 1–2 month (aOR 0.60, 95%CI 0.51, 0.72) relative to 5–6 months, fever (aOR 0.56, 95%CI 0.36, 0.88), cough or cold (aOR 0.78, 95%CI 0.66, 0.93) or pneumonia diagnoses (aOR 0.65, 95%CI 0.53, 0.81) remained significantly associated with reduced odds of having a diagnostic test result for malaria for patients with suspected malaria. No diagnosis (aOR 1.38, 95%CI 1.07, 1.78) relative to single diagnosis, a health facility in a medium—high EIR area (aOR 3.04, 95%CI 1.59, 5.78) relative to one in a very high EIR area, a health facility with clinically active staff in the 2^nd^ (aOR 1.33, 95%CI 1.01, 1.77), 3^rd^ (aOR 1.72, 95%CI 1.23, 2.39) and highest (aOR 1.88, 95%CI 1.25, 2.82) quartile relative to ones with the lowest quartile or a health facility laboratory staff in the 2^nd^ (aOR 2.58, 95%CI 1.45, 4.62) and 3^rd^ (aOR 1.60, 95%CI 1.04, 2.44) quartile relative to one with the lowest quartile remained significantly associated with increased odds of having a diagnostic test result for malaria for patients with suspected malaria.

### Antimalarial prescription for patients with a negative diagnostic test result for malaria

**Fig 4:**
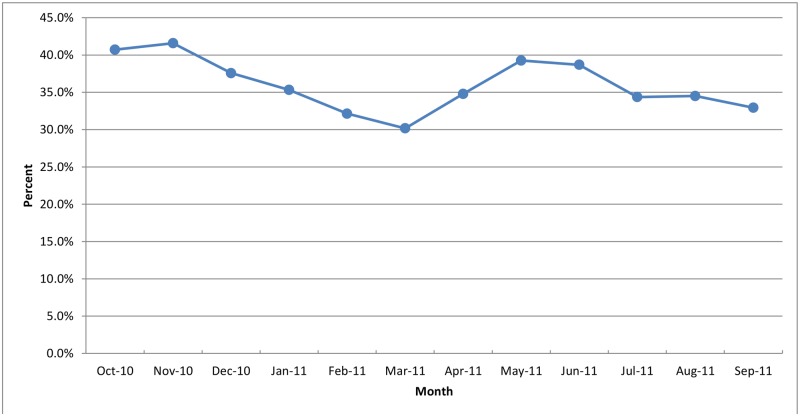
Antimalarial prescription by category of malaria diagnosis and age group in infants aged less than six in Ugandan Primary Health centers, October 2010 – September 2011.

As shown in Table 1 and Fig 1, antimalarials were prescribed for 1,545 (31.1%) of the 4,972 patients with a confirmed negative diagnostic test result for malaria. In comparison, antimalarials were also prescribed to 5,912 (55.6%) of the 10,630 patient visits with suspected malaria and no diagnostic test result for malaria recorded. In Fig 4, the percentage of visits during which patients diagnosed with malaria were prescribed any antimalarial ranged between 22.5% (age 1–2 months) and 39% (5–6 months) in patients with confirmed negative malaria test results and between 93.6% (2–3 months) and 97.1% (5–6 months) in patients with an unconfirmed malaria diagnosis.

In univariate analysis (Table 3), antimalarial prescription for patients with a negative test result for malaria was less likely if the patient was of young age (age groups 7–31 days to 4–5 months) compared to 5–6 months old; had cough/cold, diarrhea, pneumonia or UTI diagnosis; visited a health facility in a very low compared to very high EIR areas. Patient and facility factors that made antimalarial prescription for patients with a negative test result for malaria more likely were fever; anemia diagnosis; return visit; being managed by midwife or other less skilled provider compared to medical officer; visiting health facilities in medium—high compared to very high EIR areas; visiting a hospital; visiting a PNFP health facility; visiting the health facilities during shortage of antimalarials, antibiotics or ORS; or visiting a health facility with clinically active staff in 2^nd^, 3^rd^ and highest quartiles compared to the lowest quartile.

**Table 3:** Predictors of being prescribed an antimalarial when the diagnostic test result for malaria was negative among infants tested in Ugandan Primary Health centers.

	Patient with a negative test result for malaria		Univariate analysis		Multivariate Analysis	
Variable	Total (N = 4,972)	%Prescribed an antimalarial (N = 1,545)	cOR (95% CI)	P Value	aOR (95% CI)	P Value
Age group						
1–6 days	126	34.1%	0.81 (0.55, 1.19)	0.29	*0.47 (0.32, 0.69)	<0.0001
7–31 days	338	28.7%	0.62 (0.48, 0.82)	0.001	*0.38 (0.18, 0.79)	0.01
1–2 months	565	22.5%	0.45 (0.36, 0.57)	<0.0001	*0.38 (0.25, 0.56)	<0.0001
2–3 months	776	24.5%	0.51 (0.42, 0.62)	<0.0001	*0.48 (0.34,0.68)	<0.0001
3–4 months	914	27.9%	0.61 (0.50, 0.73)	<0.0001	*0.60 (0.50, 0.73)	<0.0001
4–5 months	1,109	34.9%	0.84 (0.71, 0.99)	0.04	*0.82 (0.69, 0.97)	0.02
5–6 months	1,144	39.0%	*ref*	*Ref*	*ref*	*Ref*
Fever	4,018	31.6%	1.85 (1.38, 2.49)	<0.0001	*2.22 (1.47, 3.34)	<0.0001
Cough or cold diagnosis	1,657	20.9%	0.47 (0.41, 0.53)	<0.0001	*0.39 (0.29, 0.51)	<0.0001
Diarrhea diagnosis	702	25.5%	0.73 (0.61, 0.87)	0.001	*0.62 (0.43, 0.89)	0.01
Pneumonia diagnosis	634	22.4%	0.60 (0.49, 0.73)	<0.0001	*0.35 (0.25, 0.48)	<0.0001
UTI diagnosis	259	5.8%	0.13 (0.08, 0.22)	<0.0001	*0.16 (0.07, 0.35)	<0.0001
Anemia diagnosis	33	63.6%	3.92 (1.92, 7.99)	<0.0001	*3.51 (1.42, 8.70)	0.01
Return visit	56	48.2%	2.12 (1.25, 3.60)	0.01	1.55 (0.74, 3.23)	0.24
Health provider						
Medical officer	94	20.2%	*ref*	*Ref*	*ref*	*Ref*
Clinical officer	2,506	28.5%	1.56 (0.94, 2.61)	0.08	2.01 (0.97, 4.15)	0.06
Nurse	1,130	28.5%	1.57 (0.94, 2.65)	0.09	2.13 (0.99, 4.58)	0.05
Midwife	32	40.6%	2.70 (1.14, 6.43)	0.03	*3.58 (1.08, 11.8)	0.04
Other (less skilled)	581	43.2%	3.00 (1.77, 5.10)	<0.0001	*4.75 (2.01, 11.2)	<0.0001
EIR area						
Very high	3,166	28.4%	*ref*	*ref*	*ref*	*ref*
Medium-high	1,267	40.3%	1.70 (1.49, 1.95)	<0.0001	*2.20 (1.48 3.25)	<0.0001
Low EIR	313	31.6%	1.16 (0.91, 1.50)	0.23	1.34 (0.29, 6.01)	0.71
Very low	226	15.5%	0.46 (0.32, 0.67)	<0.0001	0.43 (0.16, 1.20)	0.11
Clinically active staff quartile						
Lowest	1,063	22.9%	*ref*	*ref*	*ref*	*Ref*
2^nd^ quartile	1,370	29.6%	1.42 (1.18, 1.70)	<0.0001	1.14 (0.79, 1.65)	0.48
3^rd^ quartile	1,448	32.0%	1.59 (1.33, 1.91)	<0.0001	0.96 (0.67, 1.36)	0.80
Highest	1,091	39.7%	2.22 (1.84, 2.68)	<0.0001	1.23 (0.87, 1.74)	0.25
Hospital	864	41.3%	1.73 (1.49, 2.01)	<0.0001	*3.31 (1.10, 9.96)	0.03
Private Not for Profit	729	38.0%	1.44 (1.22, 1.69)	<0.0001	1.34 (0.57, 3.12)	0.51
Antimalarial shortage	754	48.4%	2.42 (2.06, 2.83)	<0.0001	*1.82 (1.03, 3.21)	0.04
Antibiotic shortage	2,121	39.3%	1.92 (1.70, 2.17)	<0.0001	*2.23 (1.03, 4.83)	0.042
ORS shortage	1,160	39.1%	1.59 (1.39, 1.83)	<0.0001	1.36 (0.96, 1.93)	0.09

* Statistically significant at 0.05 level.

In multivariate analysis, younger age groups 1–6 days (aOR 0.47, 95%CI 0.32, 0.69), 7–31 days (aOR 0.38, 95%CI 0.18, 0.79), 1–2 months (aOR 0.38, 95%CI 0.25, 0.56), 2–3 months (aOR 0.48, 95%CI 0.34, 0.68), 3–4 months (aOR 0.60, 95%CI 0.50, 0.73) and 4–5 months (aOR 0.82, 95%CI 0.60, 0.97) relative to 5–6 months and cough or cold (aOR 0.39, 95%CI 0.29, 0.51), diarrhea (aOR 0.62, 95%CI 0.43, 0.89), pneumonia (aOR 0.35, 95%CI 0.25, 0.48) and UTI (aOR 0.16, 95%CI 0.07, 0.35) diagnoses remained significantly associated with reduced odds of antimalarial prescription for patients with a negative test result for malaria. Fever (aOR 2.22, 95%CI 1.47, 3.34), anemia diagnosis (aOR 3.51, 95%CI 1.42, 8.70), being managed by midwives (aOR 3.58, 95%CI 1.08, 11.8) or other less skilled providers (aOR 4.75, 95%CI 2.01, 11.2) relative to medical officers, visiting a health facility in medium-high EIR area (aOR 2.20, 95%CI 1.48, 3.25) compared to one in very high EIR area, visiting a hospital (aOR 3.31, 95%CI 1.10, 9.96), and shortages of antimalarials (aOR 1.82, 95%CI 1.03, 3.21) and antibiotics (aOR 2.23, 95%CI 1.03, 4.83) were significantly associated with increased odds of antimalarial prescription for patients with a negative test result for malaria.

### Prescription of appropriate antimalarial among patients prescribed any antimalarial and weighing five or more kilograms (≥5kgs)

Over 95% of patients with an antimalarial prescription and weighing ≥5kgs received an appropriate antimalarial as per the national malaria treatment guidelines (Table 4). There was no difference in the percentage across age groups.

**Table 4:** Predictors of appropriate antimalarial prescription among infants (≥5 kgs) prescribed any antimalarial in Ugandan Primary Health centers.

Variables	Patients with ≥5kgs prescribed any antimalarial		Univariate analysis		Multivariate Analysis	
	Total (N = 3,728)	Prescribed an appropriate antimalarial (N = 3,621)	cOR (95% CI)	P Value	aOR (95% CI)	P Value
Fever	3,096	97.5%	1.86 (1.20, 2.88)	0.01	*2.52 (1.41, 4.51)	0.002
Pneumonia	520	95.6%	0.58 (0.36, 0.93)	0.02	*0.47 (0.24, 0.93)	0.03
Health Provider						
Medical officer	52	92.3%	*ref*	*Ref*	*ref*	*Ref*
Clinical officer	2,258	97.3%	3.05 (1.07, 8.74)	0.04	2.01 (0.71, 5.99)	0.18
Nurse	782	96.8%	2.52 (0.84, 7.54)	0.10	1.27 (0.31, 5.16)	0.74
Others (less skilled)	305	97.4%	3.09 (0.90, 10.7)	0.07	1.96 (0.38, 10.1)	0.43
EIR area						
Very high	3,054	97.5%	*ref*	*Ref*	*ref*	*Ref*
Medium—high	490	93.9%	0.39 (0.25, 0.60)	<0.0001	0.26 (0.06, 1.10)	0.07
Hospital	1,353	96.4%	0.66 (0.45, 0.98)	0.04	0.66 (1.15, 3.06)	0.60
Private no for Profit	605	95.7%	0.59 (0.38, 0.93)	0.02	1.75 (0.28, 11.0)	0.55

* Statistically significant at 0.05 level.

In a univariate analysis (Table 4) of factors associated with prescription of an appropriate antimalarial among patients who weighed five or more kilograms and were prescribed any antimalarial, having fever or being managed by a clinical officer compared to medical officer was significantly associated with an increased likelihood of being prescribed an appropriate antimalarial. However, pneumonia diagnosis, visiting a health facility in a medium—high EIR area compared to one in very high EIR area, visiting a hospital or visiting a PNFP health facility was associated with reduced likelihood of being prescribed an appropriate antimalarial.

In multivariate analysis, patients who weighed five or more kilograms and were prescribed any antimalarial were more likely to be prescribed an appropriate antimalarial if they had a fever (aOR 2.52, 95%CI 1.41, 4.51). On the other hand, those with a pneumonia diagnosis (aOR 0.47, 95%CI 0.24, 0.93) remained associated with reduced odds of being prescribed an appropriate antimalarial.

### Exploratory analysis of malaria diagnosis and treatment in infants weighing less than 5 kilograms (<5kgs)

**Fig 5:**
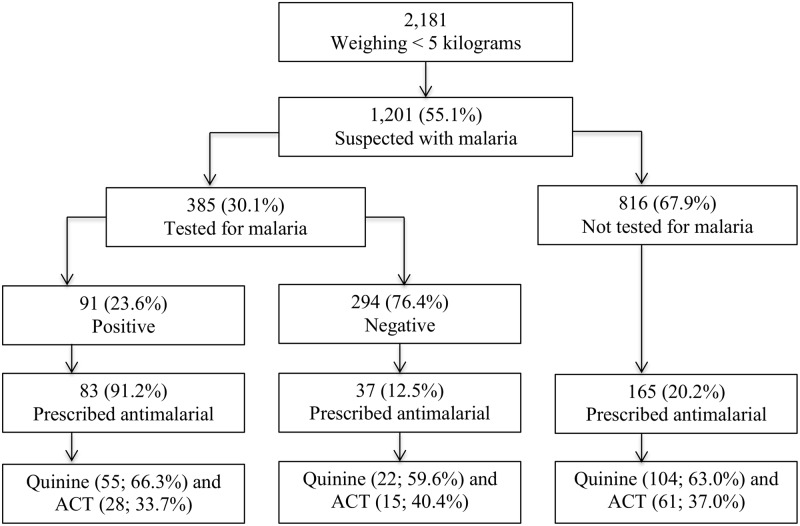
Exploratory analysis of malaria diagnosis and treatment in patients weighing <5 kilograms in Ugandan Primary Health centers, October 2010 – September 2011.

An exploratory sub analysis was conducted in a sub group of 2,181 (20.8%) patients who weighed <5kgs and results are presented in Fig 5. Malaria was suspected in 1,201 (55.1%) of these patients, of which only 385 (32.1%) had a diagnostic test result for malaria recorded and 91 (23.6%) were positive. A total of 285 patients in this group were prescribed antimalarials, but malaria was confirmed in only 83 (29.1%). Among 294 patients who had a negative test result for malaria recorded, 37 (12.5%) were prescribed an antimalarial. Of the 285 patients prescribed any antimalarial, 181 (63.5%) were prescribed quinine as per the national treatment guidelines, while 104 (36.5%) received ACT. Malaria was presumptively treated in 165 (20.2%) of the 816 patients who did not have any malaria test.

## Discussion

This is one of the first studies to measure MPPR in outpatient infants under six months old and to examine the association between clinical and operational factors and malaria care at primary health facilities among this population in Uganda. This study found high rates of malaria suspicion but low testing rates. Among those tested the MPPR was high. The study also found high rates of appropriate antimalarial prescription, but low levels of adherence to negative malaria test results.

As shown in Figs 1 and 2, 70.8% of patients aged under six months were suspected with malaria and this proportion increased with age. This rate is higher than the 64% and 59% for all outpatients reported in two Ugandan studies, Mbonye *et al*. [30] and Sears *et al*. [43] respectively.

Despite the observed high rate of malaria suspicion, only 42.3% of patients with suspected malaria had malaria test results recorded. This rate was comparable to WHO’s 2010 findings, in which proportion of malaria suspects tested in the public sector was 37% in the African region, and 44% globally [6]. Despite the WHO’s recommendation of universal testing for all suspected cases of malaria, including infants prior to antimalarial treatment [23,44], this study shows that the testing rate remains low among young infants.

As shown in Fig 1, the overall MPPR among infants tested was 36.1% and increased with age, consistent with results from a Malawi study [20]. Our estimate of patients under six months with malaria parasites is somewhat higher than health facility-based estimates reported in many other African countries [20,45,46], perhaps due in part to the use of routine, rather than expert testing [47]. The accuracy of these malaria test results was not established in this study. However, one can draw confidence in these results considering that during this study, the laboratory professionals at the health facilities were receiving on-going facility-based training and mentoring from the experienced IDCAP laboratory faculty as well as quality assurance from the Uganda’s Central Public Health Laboratory (CPHL) to improve the validity of malaria and other test results. The IDCAP laboratory training program delivered during this period was similar to an earlier one run by the Infectious Diseases Institute /Uganda Malaria Surveillance Project which successfully improved the quality of laboratory diagnosis of malaria [48]. An evaluation of the IDCAP on-site laboratory training is in progress [Burnett, personal communication]. CPHL is responsible for formulation of laboratory policy, testing guidelines and standard operating procedures. It also conducts proficiency testing and undertakes corrective action immediately or during their quarterly support supervision visits to the health facilities. Future research to determine the sensitivity and specificity of these malaria tests in similar studies and settings should be considered. Although the MPPR is not a population measure of prevalence, under certain circumstances, it was shown to be a good surrogate estimate of change in malaria incidence [49].

When parasitological results were available, overtreatment of malaria was common, with 31.1% of infants with a negative malaria test result prescribed an antimalarial. In addition, 55.6% of malaria suspects who were not tested for malaria were prescribed antimalarials, similar to studies in Kenya and Tanzania [50,51]. There is limited information on malaria treatment practices in this age group, however, malaria is widely over-treated in older age groups in Africa [52–55] which can result in failure to treat other life-threatening conditions [56]. Studies have established that withholding antimalarials for older children with negative malaria test results was generally safe [57–59], although similar information is not available for children aged under six months.

However when clinicians in our study prescribed antimalarials, they did it appropriately by following the national guidelines for 95.1% of the patients prescribed any antimalarial. Considering that only between 16 to 41% of the overall total number of children with *P*. *falciparum* who in 2013 were brought for care at public health facilities in sub-Saharan Africa received ACT [6], our results showed remarkably better performance. This could be in part due to the IDCAP training interventions that improved health workers’ performance in prescribing antimalarials appropriately [30].

WHO has established guidelines for ACT prescription in children weighing ≥5kgs [23]. The Uganda Ministry of Health prohibit prescription of ACT for uncomplicated malaria to children under four months or weighing <5kgs and recommends quinine for their malaria treatment [24]. Our study established that quinine was prescribed to 63.5%, but interestingly, off-label ACT was widely prescribed to 36.5% of the patients prescribed antimalarials and weighing <5kgs.

The high rates of malaria suspicion and MPPR and low rates of testing highlight the challenges that clinicians face when treating infants under six months. In order to better understand clinician practice amidst limited information on ACT use and resulting treatment outcomes in infants under six months, clinical and operational predictors of malaria care were established.

Being of a much younger age group reduced the odds of malaria testing and antimalarial prescription for patients with negative malaria test results. Infants under six months are perceived to have protection against malaria, but this protection is thought to wane as the baby grows [11,60,61], findings that were corroborated by this study in which the MPPR increases from less than 24% in children under two months to 42.8% in 5–6 months age group. This perceived protection may explain health workers’ reluctance to conduct malaria tests for younger infants. It may also explain the higher rates of antimalarial prescription to older infants with negative malaria test results in our study, similar to findings in other studies with older patients [50,51]. Clinicians may feel more comfortable withholding antimalarials in younger infants with negative malaria test results if they believe that they are less likely to have malaria.

This study also found that fever was associated with significantly lower odds of malaria testing for suspects and higher odds antimalarial prescription for patients with negative malaria test results and appropriate antimalarial prescription. In Uganda where fever connotes malaria [62], it is possible that clinicians are more likely to believe that children with fever have malaria and do not feel they need a test to confirm it. Similarly, clinicians may not be convinced by a negative test result in fever cases and will prescribe antimalarials regardless of test results.

As in Malawi and Kenya reports, our study showed that medical doctors were more likely than lower level health providers to withhold antimalarials from patients with a negative malaria result [54,63]. Due to their higher level of training, medical doctors may be better equipped and more confident to withhold antimalarials and also diagnose alternative diseases when faced with a negative malaria test result. In our study, it was also observed that better staffing of health facilities increased the odds of testing for malaria suspects. In other studies, better staffing translated into better quality of care [64–66].

In this study, less missing data were observed compared to what has been reported in similar busy health centers elsewhere [67]. This, however, does not mean that these data were without limitations. Some checkboxes may not have been ticked during data collection, resulting in underestimation of some of the results. Also, it was not possible to control for multiple visits by the same patient, because the data were anonymous and patients did not have unique identifiers. The accuracy of the diagnostic tests used was also not established. In this study, we did not collect data on laboratory reagents and how often the laboratory equipment broke down which might have potentially influenced the testing rates and the results of the regression models. Considering this was a health facility based study, the choice of variables to include in the study was limited to the patient and a few health facility characteristics. Future studies might consider collecting data on other variables, such as laboratory stocks, that could have been important but were not included in this study. Also, an analysis on dosage and toxicity of ACT and other antimalarials prescribed was not done, neither was a follow-up of patients to establish the outcomes of the treatment.

## Conclusion

This study found high rates of malaria suspicion and appropriate antimalarial prescription in infants under six months old and weighing at least 5kg. The study also found low rates of testing, high MPPR among patients tested, and moderate rates of antimalarial prescription among those with a negative malaria test result in this population. Off-label prescription of ACT for infants weighing <5kgs was common despite lack of evidence on ACT effectiveness in this young population and the Uganda malaria treatment guidelines, which prohibit their use.

Malaria is the single biggest contributor of infant mortality in Uganda and hence, better malaria care practices are essential in order to save the lives of this most-at-risk population. Further research should be done to test the efficacy and toxicity of antimalarials and accuracy of malaria diagnostic techniques currently used in infants under six months old, and to find new drug candidates for malaria control. Evidence-based malaria guidelines for infants weighing under five kilograms and aged under six months are urgently needed.
